# Roles of the Amino Group of Purine Bases in the Thermodynamic Stability of DNA Base Pairing

**DOI:** 10.3390/molecules190811613

**Published:** 2014-08-05

**Authors:** Shu-ichi Nakano, Naoki Sugimoto

**Affiliations:** 1 Department of Nanobiochemistry, Faculty of Frontiers of Innovative Research in Science and Technology (FIRST), Konan University, 7-1-20, Minatojima-minamimachi, Chuo-ku, Kobe 650-0047, Japan; 2 Frontier Institute of Biomolecular Engineering Research (FIBER), Konan University, 7-1-20, Minatojima-minamimachi, Chuo-ku, Kobe 650-0047, Japan; E-Mail: sugimoto@konan-u.ac.jp

**Keywords:** DNA oligonucleotide, Watson-Crick base pair, hydrogen-bonding amino group, 2'-deoxyriboinosine, 2'-deoxyribo-2,6-diaminopurine, thermodynamic parameters, complementary base recognition, ion binding

## Abstract

The energetic aspects of hydrogen-bonded base-pair interactions are important for the design of functional nucleotide analogs and for practical applications of oligonucleotides. The present study investigated the contribution of the 2-amino group of DNA purine bases to the thermodynamic stability of oligonucleotide duplexes under different salt and solvent conditions, using 2'-deoxyriboinosine (I) and 2'-deoxyribo-2,6-diaminopurine (D) as non-canonical nucleotides. The stability of DNA duplexes was changed by substitution of a single base pair in the following order: G•C > D•T ≈ I•C > A•T > G•T > I•T. The apparent stabilization energy due to the presence of the 2-amino group of G and D varied depending on the salt concentration, and decreased in the water-ethanol mixed solvent. The effects of salt concentration on the thermodynamics of DNA duplexes were found to be partially sequence-dependent, and the 2-amino group of the purine bases might have an influence on the binding of ions to DNA through the formation of a stable base-paired structure. Our results also showed that physiological salt conditions were energetically favorable for complementary base recognition, and conversely, low salt concentration media and ethanol-containing solvents were effective for low stringency oligonucleotide hybridization, in the context of conditions employed in this study.

## 1. Introduction

DNA is a molecule with the ability to assemble base pairs through the formation of hydrogen bonds. The G•C base pair forms three hydrogen bonds, and the A•T base pair forms two hydrogen bonds, leading to a greater thermodynamic stability of G•C than A•T base pairs [1]. A wobble G•T pair also forms two hydrogen bonds with a geometry similar to that of Watson-Crick base pairs, resulting in a stability comparable to that of an A•T base pair [2]. The energetic aspects of base pair formation are important for the creation of secondary and higher-order structures and the practical application of oligonucleotides for hybridization-based detection and targeting assays. However, the small differences between the interaction energies of complementary and mismatched pairs cause a difficulty in discriminating a target sequence. The interaction energy arising from the hydrogen bonding of a group of nucleobases can be experimentally evaluated using non-canonical bases that lack or have additional hydrogen-bond donating or accepting groups. For example, there are reports indicating the use of inosine, an analog that lacks the 2-amino group of guanine but can pair with cytosine through two hydrogen bonds in the same geometry as the G•C base pair. Substitution of inosine for guanine in a base pair with cytosine has been found to decrease the thermodynamic stability of oligonucleotide duplexes [3,4], and the extent of the effect of the substitution in DNA (decreases in −∆*G*° by 1.7 kcal∙mol^−^^1^ or less) was reported to be smaller than the case of RNA duplexes [5]. It was also reported that substitution of 2,6-diaminopurine for adenine that created an additional hydrogen bond with thymine enhanced the stability of DNA duplexes (increases in −∆*G*° by ~1 kcal∙mol^−^^1^ or less) [6,7]. There have also been computational studies of the contributions of hydrogen-bonding groups to base-pair stability. Although many of the studies considered the gas-phase interaction energy of DNA bases, possible factors in addition to the primary hydrogen-bonding interactions have been suggested to contribute to the base-pair stability, such as cross-hydrogen-bonding between frontier atoms of base pairs [8], C-H-O contacts [9,10], and electrostatic interactions between distant atoms [11,12]. However, the additional contribution of hydrogen-bonding groups to base-pair stability, besides complementary base-pair formation through hydrogen bonds, remains to be investigated.

DNA duplex stability is strongly dependent on the salt concentration and solvent conditions. It was previously found that the effect of the substitution of inosine for guanine in DNA was not the same under conditions of moderate and low salt concentrations [5]. In an effort to determine the electrostatic component of the 2-amino group of DNA purine bases, the present study compared the thermodynamic parameters of DNA duplexes under different salt and solvent conditions, using 2'-deoxyriboinosine (I) and 2'-deoxyribo-2,6-diaminopurine (D) as non-canonical nucleotides. This study was designed to investigate the role of the 2-amino group of purine bases in determining the stability of DNA duplexes and the salt effects, which have not been well addressed. Our results show that the 2-amino group of G and D provides a large amount of stabilization energy, but the amount varies depending on the salt and solvent conditions that arise when using Na^+^, Mg^2+^, and ethanol-containing solvent. Sequence-dependent salt effects on the thermodynamic stability of DNA duplexes are presented, and the role of the 2-amino group in affecting ion binding as well as in creating base-pair hydrogen bonding is proposed. The results of this study provide insight into the design of non-natural nucleotides and suggest suitable experimental conditions for practical applications of DNA oligonucleotides.

## 2. Results and Discussion

### 2.1. DNA Duplexes in Solutions Containing Na^+^ of Various Concentrations

**Figure 1:**
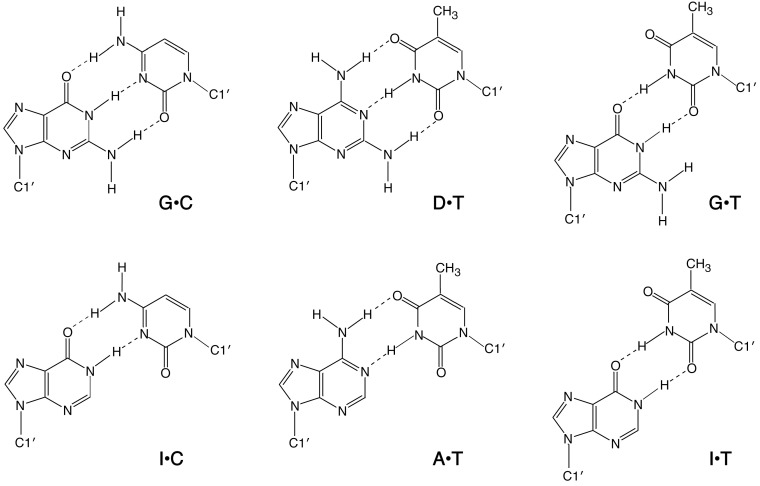
Base-paired structures of X•Y in the DNA duplexes used in this study. Dotted lines represent hydrogen bonds, and C1' represents the anomeric carbon of the sugar moiety of DNA.

We prepared six kinds of 13-mer DNA duplexes with the sequence 5'-TTTGTATCXCAAT-3'/5'-ATTGYGATACAAA-3'. These duplexes have the same primary sequence except the single internal X•Y pair, which is of either G•C, I•C, D•T, A•T, G•T, or I•T. These base-paired structures are compared in Figure 1. The G•C and D•T base pairs form three hydrogen bonds and the I•C and A•T base pairs form two hydrogen bonds. In the G•C and D•T pairs, the 2-amino group of G and D forms a hydrogen bond with a complementary pyrimidine nucleotide. The atoms exposed in the minor groove of G•C and D•T base pairs are the same, but the positions of atoms exposed in the major groove are different. Similarly, I•C and A•T base pairs have the same minor groove but different major groove faces. It must be emphasized that I•T forms two hydrogen bonds, as does G•T, because inosine lacks the amino group that does not form a hydrogen bond with thymine.

**Figure 2:**
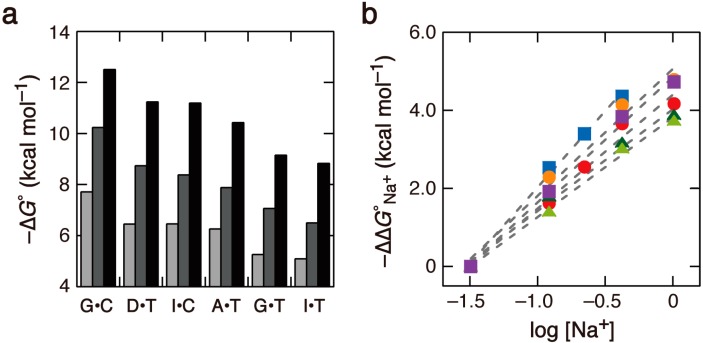
(**a**) Comparison of the values of −∆*G*° for duplex formations in solutions containing 30 mM (light gray), 120 mM (dark gray), and 1 M Na^+^ (black); (**b**) Differences in ∆*G*° of the duplexes with G•C (cyan), I•C (purple), D•T (orange), A•T (red), G•T (green), and I•T (yellowish green), represented by −∆*G*°_Na_^+^ (= ∆*G*°_30 mM_ − ∆*G*°_obs_), at different Na^+^ concentrations.

The energy values apparently arising from the 2-amino group of G and D were evaluated from the thermodynamic parameters for the formation of DNA duplexes, determined through the ultraviolet (UV) melting curves analysis. The thermal melting curves of DNA duplexes were measured in solutions containing varying concentrations of Na^+^ (Supplementary Table S1). Thermodynamic parameters determined from a plot of *T*_m_^−1^ *vs.* log (*C*_t_/4), which was derived from the melting temperature (*T*_m_) at each strand concentration (*C*_t_), were similar to the parameters determined by fitting of the melting curves, which is indicative of two-state thermal denaturation. The duplex stability, defined as the value of −∆*G*° extrapolated to 37 °C, was different depending on the type of X•Y: the highest stability was found for the duplex with G•C and the lowest stability was found for the duplex with I•T (Figure 2a). The duplex with I•C had a lower stability than the duplex with G•C, and the duplex with D•T had a higher stability than the duplex with A•T, as reported in previous studies [4,5,6,7]. These results indicate the significance of 2-amino groups for base-pair stability. Based on the thermodynamic parameters of the duplex with G•T, values of −∆*G*°, −∆*H°*, and −∆*S°* that result from formation of the CGC/GTG trinucleotide pairs, calculated as previously described [13], were 0.7 kcal∙mol^−^^1^, 10.0 kcal∙mol^−^^1^, and 29.5 cal∙mol^−^^1^∙K^−1^, respectively at 1 M Na^+^. These values are similar to the values predicted using the nearest-neighbor interaction model, in which NMR spectroscopy confirmed the formation of two hydrogen bonds between G and T, which are 1.06 kcal∙mol^−1^ for −∆*G*°, 8.5 kcal∙mol^−1^ for −∆*H°*, and 23.0 cal∙mol^−1^∙K^−1^ for −∆*S°* [2]. The duplex with I•T showed slightly, but significantly lower stability than the duplex with G•T, whereas the number of interbase hydrogen bonds was the same. The duplex stabilities, measured as −∆*G*° at 1 M Na^+^, were in the following order: G•C (12.5 kcal∙mol^−1^) > D•T (11.2 kcal∙mol^−1^) ≈ I•C (11.2 kcal∙mol^−1^) > A•T (10.4 kcal∙mol^−1^) > G•T (9.15 kcal∙mol^−1^) > I•T (8.83 kcal∙mol^−1^). This order remained the same over the Na^+^ concentration range from 30 mM to 1 M (Figure 2a). However, the extent of the change in −∆*G*° by increasing the Na^+^ concentration from 30 mM was different among the duplexes (Figure 2b). Owing to enthalpy-entropy compensation phenomena and the small experimental error in ∆*G*° [14], the differences among the duplexes are substantial.

Regardless of the type of X•Y, the duplexes showed similar circular dichroism (CD) spectra, with a positive band at 280 nm and a negative band at 250 nm. Although the spectra are not particularly sensitive to subtle or local conformational changes, it is probable that the nucleotides X and Y adopted a conformation that did not alter the overall B-form of the DNA helix. These spectra were also insensitive to the salt concentration and solvent composition investigated in this study (Figure S1). The CD spectral data suggested that the differences in thermodynamic stabilities among the duplexes resulted from different interactions near the X•Y base pair.

The difference in thermodynamic parameters between the duplexes with G•C and I•C or between the duplexes with D•T and A•T was attributed to the interaction energy due to the presence of the 2-amino group of G or D, respectively. The hydrogen-bonding amino groups increased the duplex stability by 1.3–1.9 kcal∙mol^−1^ (differences in ∆*G*° between the duplexes with G•C and I•C) and 0.2–0.9 kcal∙mol^−1^ (differences in ∆*G*° between the duplexes with D•T and A•T), originated from the ∆*H*° term, with the variation depending on the salt concentration. These results indicate that the apparent stabilization energies arising from the 2-amino group of G and D are highly salt concentration-dependent. Furthermore, the duplex with G•T had 0.2–0.4 kcal∙mol^−1^ higher stability compared to the duplex with I•T, despite the fact that inosine lacks the non-hydrogen-bonding amino group. Therefore, the number of hydrogen bonds in the X•Y pair does not fully explain the stability order of the duplexes, although the amino groups that form base-pair hydrogen bonding provide a large amount of stabilization energy.

### 2.2. Duplex Stabilities in Water-Ethanol Mixed Solvents and Mg^2+^ Solutions

**Figure 3:**
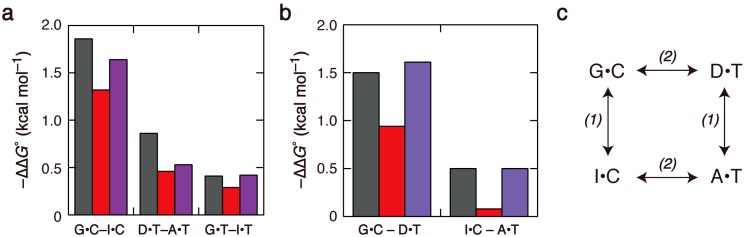
(**a**,**b**) Differences in ∆*G*° between duplexes that do or do not have the 2-amino group (**a**) and those between duplexes that have different major groove faces (**b**), measured in solutions containing 120 mM Na^+^ (black), 120 mM Na^+^ with 3 M ethanol (red), or 10 mM Mg^2+^ (purple); (**c**) The relationship among the base-paired structures of X•Y in the investigated duplexes: (1) represents a comparison between the base pairs having different minor groove atoms; and (2) represents a comparison between those that have different positions of the major groove atoms.

Duplex stability changes with differences in solvent composition as well as salt concentrations [15,16]. The thermodynamic effects of water-soluble organic compounds on nucleic acids have been studied from the point of view of molecular crowding, and significant effects arising from water activity and the dielectric constant of solutions on the stability of oligonucleotide structures have been reported [17]. Water-ethanol mixed solvent at a concentration of 3 M was examined in this study. Ethanol is a protic solvent having hydrogen-bond donating and accepting capabilities that could compete with DNA base-pair hydrogen bonding. Furthermore, ethanol solutions have properties that differ from those of a pure aqueous solution, such as water activity, dielectric constant, viscosity, and dipole moment. Because the *T*_m_ values of the duplexes in ethanol-containing solvents were not very high, no significant evaporation occurred during thermal melting of the DNA. The presence of ethanol in the solution decreased the stability of each duplex, compared with their stability in a solution lacking ethanol, but the stability order remained the same. The decrease mostly originated from the ∆*S*° term (Table S2). The differences in ∆*G*° between G•C and I•C, between D•T and A•T, and between G•T and I•T became smaller in the mixed solvent (Figure 3a). This finding indicates that the apparent stabilization energy arising from the presence of the 2-amino group of G and D decreased in the presence of ethanol. We also examined solutions containing Mg^2+^. The duplex stabilities were high with relatively low amounts of Mg^2+^, and similar ∆*G*° values were obtained with 10 mM Mg^2+^ and 400 mM Na^+^ (Table S3). The duplex stability order in Mg^2+^ solution was similar to that in Na^+^ solution, and again the 2-amino group of G provided greater stabilization energy than the 2-amino group of D (Figure 3a).

It is not appropriate that the differences in stability between the duplexes containing G•C and I•C and between the duplexes containing D•T and A•T are attributed exclusively to the interaction energy of base-pair hydrogen bonding. This interaction can be balanced with other energy contributions and affected by a cooperative network of interactions [3,18,19]. Non-canonical hydrogen bond interactions and electrostatic interactions between distant atoms of DNA bases have also been reported to be important [8,9,11,12]. Our observation of higher duplex stability with G•C than with D•T base pairs is consistent with the significance of secondary hydrogen-bonding interactions caused by different arrangements of the donor and acceptor groups of triply hydrogen-bonded base pairs [8,20]. Deletion or addition of an amino group further changes the distribution of electrons at the surface of purine bases and the strength of stacking interactions [10]. In addition to these contributions, our results suggest that there are other roles for the amino groups that cause salt and solvent-dependent effects on duplex stabilities.

The DNA duplexes investigated in this study have different helical groove faces. G•C and I•C base pairs and D•T and A•T base pairs have different atoms in the minor groove; and the presence of the 2-amino group of G or D do or do not result in widened minor groove and could affect the elastic property of DNA duplex [21,22,23], while no significant change in the CD spectra was observed (Figure S1). The differences in ∆*G*° between G•C and I•C and those between D•T and A•T base pairs, denoted by (1) in Figure 3c, are presented in Figure 3a. The stabilization energy arising from the hydrogen bonding of the 2-amino group of G was largely different from that arising from the hydrogen bonding of the 2-amino group of D, both in Na^+^ and Mg^2+^ solutions, and their stabilization energies decreased in the 3 M ethanol solution. A comparison of G•T and I•T base pairs also showed increased interaction energy due to the presence of the 2-amino group of G. To compare with the effects of the change in the positions of major groove atoms, the differences denoted by (2) in Figure 3c were also analyzed. The data in Figure 3b show that the differences between G•C and D•T base pairs were greater than those between I•C and A•T base pairs, and that the differences decreased in ethanol solution, but less in Mg^2+^ solution. It is notable that the energy changes resulting from the substitution of the major groove atoms were comparable to those resulting from removal of the hydrogen-bonding amino group. These findings suggest that the groove atoms significantly contribute to the base pairing energy, in addition to the formation of base-pair hydrogen bonding, which could be heavily influenced by the presence of ethanol.

### 2.3. Comparison of the Salt Concentration Dependences

The present study used base-paired duplex sequences in which atmospherically bound ions play a central role in determining the duplex stability. A simplified polyelectrolyte model considers sequence-independent ion binding and provides a qualitative interpretation of DNA-ion interactions [24,25,26]. In this model, the extent of the salt concentration dependence is determined by the number of phosphates and the charge density along a DNA helix. Therefore, the effects of salt on the thermodynamic stability of duplexes of the same length are predicted to be the same. However, our results disagreed with this prediction. The thermodynamic degree of ion accumulation near nucleic acids determines the effect of salt concentration on the duplex stability [14,27]. Analysis of the dependence of −∆*G*° on the logarithm of the ion concentration (log [M]) assumes ion binding to defined sites according to the stoichiometric mass-action law, but it at least qualitatively provides the degree of ion binding to DNA that affects thermodynamic stability [28]. The linear correlations given in Figure 2b provide sequence-dependent slope values (Table 1), that indicate net binding ranging from 3.1 Na^+^ ions for G•C base pairs to 2.1 Na^+^ ions for I•T base pairs, although the numbers averaged per phosphate fell within the range of those reported for other sequences [14,27,29,30,31,32]. The analysis used the ∆*G*° values extrapolated to a certain temperature, 37°C in this case. Nevertheless, the ion binding numbers were approximately the same as those determined using an alternative method that analyzed the dependence of *T*_m_^−1^ on log [Na^+^] [14,33]. These values ranged from 2.9 (for G•C) to 2.3 (for I•T).

**Table 1:** Dependence of −∆*G*° of the duplexes on log [M] in a linear range under different solution conditions.

X•Y pair	Na^+^ solution	Na^+^ with 3 M ethanol solution	Mg^2+^ solution
G•C	3.91 ± 0.19	3.32 ± 0.09	2.63 ± 0.26
D•T	3.25 ± 0.32	3.17 ± 0.21	2.00 ± 0.22
I•C	3.21 ± 0.16	3.19 ± 0.11	2.15 ± 0.16
A•T	2.91 ± 0.23	2.74 ± 0.09	1.92 ± 0.19
G•T	2.61 ± 0.18	2.43 ± 0.17	1.59 ± 0.26
I•T	2.55 ± 0.13	2.41 ± 0.13	1.50 ± 0.26

The numbers of Na^+^ ions bound in the ethanol-containing solution were calculated to be in the range of 2.6 (for G•C)–1.9 (for I•T), using the sequence-dependent slope values shown in Table 1. The experimental uncertainties were not low enough, but the numbers appeared to be slightly smaller than those obtained in the solution without ethanol. A previous computer simulation study reported that the dielectric constant effect enhanced electrostatic DNA helix-helix attraction with a smaller increase in the number of bound ions [34]. In addition, we recently found significance of the dielectric constant of a mixed solvent to the salt concentration dependence of the stability of a short RNA duplex [35]. Therefore, it can be argued that the low dielectric constant environment created by ethanol changes the efficiency of the accumulation of Na^+^ on DNA. A reduced dielectric constant strengthens electrostatic interactions and increases the degree of ion binding to single-stranded as well as duplex DNA molecules. Thus, a greater degree of ion accumulation on structurally flexible single strands can explain the decreased numbers of bound Na^+^ ions during the transition from single strands to duplexes in the ethanol-containing solvent. In addition, the numbers of bound Mg^2+^ (2.1 for G•C to 1.2 for I•T) calculated from the dependence of ∆*G*° on log [Mg^2+^] (Table 1), were smaller than those obtained in Na^+^ solution, in agreement with the requirement for a smaller number of Mg^2+^ to shield the charge density of the phosphate groups along the DNA backbone. The results indicate that the X•Y pair also influenced the Mg^2+^-binding property of DNA.

Metal ions primarily associate with DNA phosphate groups, and the bases that create electronegative pockets in the helical grooves are also the potential ion-binding sites. High-resolution crystal structures [36,37,38], NMR spectroscopy [39], and molecular dynamics calculations [40,41,42,43,44,45] have revealed the presence of monovalent and divalent cations located in the major and minor grooves. Specifically, the minor groove of AT-tract sequences in a DNA duplex preferentially accommodates monovalent cations [39,43]. However, the duplex sequences used in this study do not have an AT-tract near the X•Y pair. Other specific cation binding sites are the helical groove atoms of G•C pairs. High-resolution crystal structures of B-DNA dodecamers revealed that Mg^2+^ ions bound to the major groove of a GG/CC dinucleotide step and to the minor groove of a GC/GC step [37]. Computer simulation studies also showed hydrated Mg^2+^ ion bound to the *N*7 of guanine [45]; this interaction was suggested to affect the strength of base pairing with cytosine, possibly by influencing interbase hydrogen bonding, tautomeric equilibria of bases, and cation-π interactions involving nucleobases [10,46]. The presence of specific binding sites at G•C base pairs seems to explain our observation that the stability of the duplex with a G•C base pair had a greater salt concentration dependence than those of other duplexes. However, the replacement of G•C by I•C (causing a change in the minor groove atoms) largely reduced the dependence on the concentrations of Na^+^ and Mg^2+^, as much as the replacement of G•C by D•T (causing a change in the major groove atoms) (Table 1). On the other hand, the replacement of A•T by D•T (causing a change in the minor groove atoms) and the replacement of A•T by I•C (causing a change in the major groove atoms) caused relatively small changes. It is therefore unlikely that the groove atoms directly determine the extent of the salt concentration dependence. In contrast, it appears that the greater slope values indicated in Table 1 are found for more stable duplexes. It was found that there exists a correlation between the values of the slope and the y-intercept of the linear regression of the dependence of −∆*G*° on log [M] (Figure S2). Because the y-intercept value represents the −∆*G*° at an ion concentration extrapolated to 1 M (log [M] = 0), this correlation suggests the relevance between the number of ion binding and the thermodynamic stability of duplexes with a similar sequence, regardless of whether the 2-amino group forms interbase hydrogen bonds or whether the ethanol that specifically interacts with DNA molecules was included in the solution. Because stable duplexes decrease structural flexibility and prevent base pairs from opening, they may have increased retention of ions on their surfaces.

### 2.4. Significance of Sequence-Dependent Salt Effects on Oligonucleotide Hybridization

**Figure 4:**
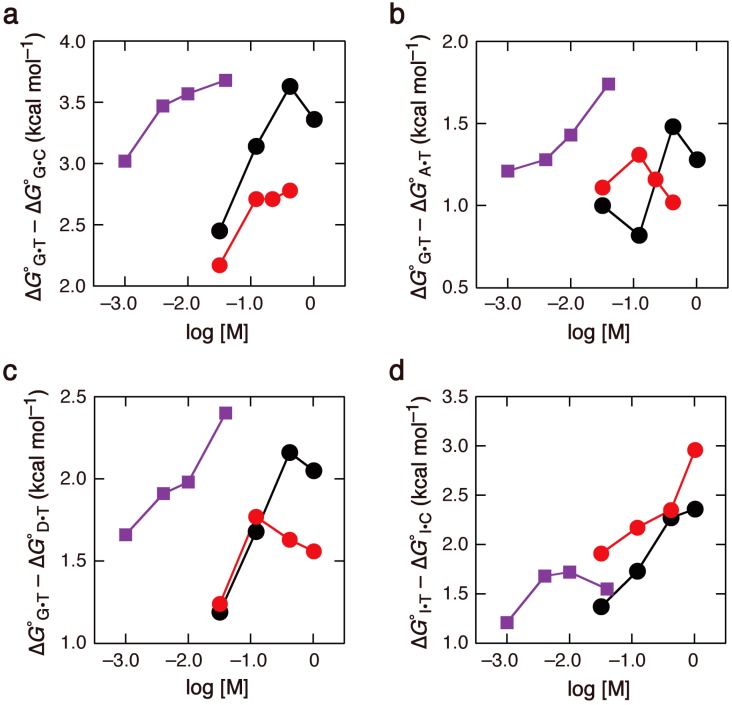
Differences in ∆*G*° between the duplexes with G•C and G•T base pairs (**a**); those between the duplexes with A•T and G•T base pairs (**b**); those between the duplexes with D•T and G•T base pairs (**c**); and those between the duplexes with I•C and I•T base pairs (**d**), at different concentrations of Na^+^ (black), Na^+^ in ethanol solution (red), or Mg^2+^ (purple).

The salt effect can be extended to practical applications of oligonucleotide hybridization. Minimizing the formation of mismatched base pairs is important for detecting the genotype of single nucleotide polymorphism (SNP) in a genome, which is based on the difference in stability between complementary and mismatched pairs formed with a DNA probe [47,48]. Discrimination of G•C or A•T pairing from wobble G•T pairing is particularly important because G•T is one of the most stable non-Watson-Crick pairs. Figure 4a shows a comparison of the differences in ∆*G*° of the duplexes with G•C and G•T under different solution conditions. Due to the sequence-dependent effect of the concentration of salt, a larger difference in ∆*G*° was observed at high, physiological salt concentrations. It was also shown that ethanol solution did not provide an obvious advantage to increase the difference. These results were also consistent when the differences between the duplexes with A•T and G•T or the differences between the duplexes with D•T and G•T were compared (Figure 4b,c), although some data at very high salt concentrations, such as 1 M NaCl, showed decreased differences, due to saturation of the effect of salt concentration on duplex stability. Based on the results obtained in the context of conditions employed in this study, solution conditions containing physiological ionic strengths of Na^+^ and Mg^2+^ have a lower probability of G base pairing with T and are suitable for SNP detection. Another possible application of the sequence-dependent salt effect is to increase the effectiveness of degenerate hybridization probes. In these probes, I and D reduce the discriminating nature of the hybridization probes among ambiguous bases, and reduce the number of primers needed for the determination of an mRNA sequence or other templates with ambiguous sequences [7,49]. The differences in stability between the duplexes with I•C or I•T shown in Figure 4d as well as the results in Figure 4a–c suggest that low ionic strength is suitable for the use of degenerate hybridization probes for target DNA sequences.

**Figure 5:**
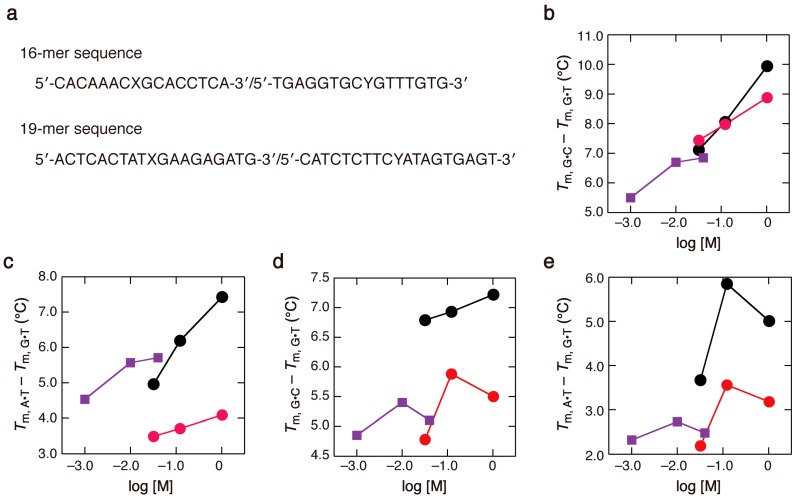
(**a**) DNA sequences of the 16-mer and 19-mer duplexes (X•Y = G•C, A•T or G•T); (**b**–**e**) Differences in the *T*_m_ values of 16-mer duplexes (**b**,**c**) and 19-mer duplexes (**d**,**e**) at strand concentrations of 2 µM, at different concentrations of Na^+^ (black), Na^+^ with 3 M ethanol (red), or Mg^2+^ (purple).

We further investigated complementary base recognition in DNA sequences of 16 or 19 nucleotides containing either G•C, A•T, or G•T base pairs (Figure 5a). Such lengths are often used for hybridization-based detection of target DNA sequences, but do not commonly allow analysis of thermodynamic parameters from the thermal melting curves, due to a non-two-state transition profile. The differences in *T*_m_, indicating the midpoint temperature of the absorbance transition between the duplexes with G•C and G•T base pairs (Figure 5b,d) and those between the duplexes with A•T and G•T base pairs (Figure 5c,e), became greater at physiological salt concentrations, as found with the 13-mer duplexes. Based on these results, it can be concluded that, regardless of the type of adjacent base pairs of the X•Y pair, target base recognition through Watson-Crick base pairing becomes more stringent at high salt concentrations, less stringent at low salt concentrations, and, in many cases, less stringent in ethanol-containing solvent. Previously, we found less effective discrimination between matched and mismatched bases when using a solution containing poly(ethylene glycol) with an average molecular weight of 200 [50]. The use of high molecular weight compounds might provide additional information about solvent effects, although such agents facilitate the precipitation of DNA during thermal melting [35]. Further comprehensive studies on the thermodynamic behavior of sequence- and solvent-dependent salt effects and considering the effect of solution pH will be useful for choosing suitable experimental conditions for oligonucleotide hybridization.

## 3. Experimental Section

### 3.1. Materials and Buffers

Oligonucleotides with 13-mer sequences were chemically synthesized on solid supports using the phosphoramidite method (Applied Biosysmtems DNA Synthesizer, Carlsbad, CA, USA). They were purified using reversed-phase HPLC, and desalted with a C18 Sep-Pak cartridge (Waters, Milford, MA, USA). Oligonucleotides with 16-mer or 19-mer sequences were purchased from Fasmac Co. Ltd. (Atsugi City, Japan). The DNA strand concentration was determined from the absorbance at 260 nm using an extinction coefficient calculated from the extinction coefficients of mononucleotides and dinucleotides [51]. DNA duplexes were prepared by mixing equimolar concentrations of complementary DNA strands.

Sodium ion solutions were made with sodium chloride, 10 mM sodium phosphate, and 1 mM EDTA at pH 7.0, and Mg^2+^ solutions was made with magnesium chloride and 10 mM sodium cacodylate at pH 7.0. Ethanol-containing solvents were prepared by mixing HPLC grade ethanol and distilled water; salts were added before the pH was adjusted to 7.0. All reagents used to prepare the buffer solutions were purchased from Wako (Osaka, Japan), except for EDTA, which was obtained from Dojindo (Tokyo, Japan).

### 3.2. CD Spectra and UV Melting Curves

CD spectra were obtained using a spectropolarimeter (JASCO J-600, Japan) equipped with a temperature controller. The cuvette-holding chamber was flushed with a constant stream of dry N_2_ gas. The spectra were measured at a DNA strand concentration of 70 µM in a 1 mm-path length cuvette.

UV absorbances were measured with a spectrophotometer (Hitachi U-3210, Japan or Shimadzu UV-1800, Tokyo, Japan) equipped with a temperature controller. Water condensation on the exterior of the cuvette in the low-temperature range was avoided by flushing the cuvette chamber with a constant stream of dry N_2_ gas. DNA melting curves were obtained at 260 nm, using a heating rate of 0.5 °C∙min^−1^ for measurements in a 1 mm path length cuvette, and a heating rate of 1.0 °C∙min^−1^ for measurements in a 10 mm path length cuvette. Each melting curve was fitted to a theoretical two-state transition curve to derive the thermodynamic parameters of ∆*H*°, ∆*S*°, and ∆*G*° at 37 °C, using a nonlinear least-squares algorithm [52]. We also analyzed the plot of *T*_m_^−1^ *vs*. log (*C*_t_/4) [53]. The thermodynamic parameters used for the comparisons were the average values obtained from curve fitting and the plot of *T*_m_^−1^ *vs*. log (*C*_t_/4).

### 3.3. Analysis of the Number of Ions Bound during Duplex Formation

The DNA-binding properties of ions were evaluated from the dependence of duplex stability, as ∆*G*° or *T*_m,_ on the salt concentration, in which duplex formation is induced by ion binding that screens DNA phosphate charges. The number of ions bound during the transition from single strands to a duplex (∆*n*) was calculated using the equation δ(−∆*G*°)/δlog [M] = 2.303α*RT*∆*n*, where *R* is the gas constant and *T* is the absolute temperature. The factor *α*, which accounts for nonideality of the salt solutions, was considered to be 0.9 for experiments using NaCl, and 0.88 for experiments using MgCl_2_ [14,27]. The value of ∆*n* was also obtained from the slope of the plot of *T*_m_^−1^ *vs.* log [M] on the basis of the equation δ(*T*_m_^−1^)/δlog [M] = 2.303α*R*∆*n*/∆*H*°, where ∆*H*° was assumed to be constant over the salt concentration range [14,33].

## 4. Conclusions

The present study showed that the apparent stabilization energy arising from the hydrogen bonding of the 2-amino group of G was largely different from that arising from the hydrogen bonding of the 2-amino group of D. The energy changes resulting from the substitution of the major groove atoms were also significant and comparable to those resulting from the addition or removal of the hydrogen-bonding amino groups. These stabilization energies varied depending on the concentration of salt and decreased in the ethanol-containing solvent. We also found that the effect of salt concentration on the thermodynamic stability of DNA duplexes was different among duplexes having substitution of a single base pair. Analysis of the salt concentration dependence suggested that the 2-amino group of the purine bases might have an influence on the binding of ions to DNA through the formation of a stable base-paired structure. These findings are useful for designing non-natural, functional nucleotide analogs and provide a valuable insight into suitable experimental conditions for practical applications of DNA oligonucleotides.

## Supplementary Materials

Supplementary materials can be accessed at: http://www.mdpi.com/1420-3049/19/8/11613/s1.

## Supplementary Files

Supplementary File 1
