# Supplementary material for: Roles of the Amino Group of Purine Bases in the Thermodynamic Stability of DNA Base Pairing
Source: Molecules. 2014 Aug 5;19(8):11613–27. doi: 10.3390/molecules190811613 (PMC6271411; doi:10.3390/molecules190811613)

Supplementary Information

Table S1. Thermodynamic parameters of DNA duplexes in solutions of varying Na^+ concentration.

**X/Y**	**[Na^+] (mM)**	**$-\Delta H°$ (kcal·mol^{-1})**	**$-\Delta S°$ (cal·mol^{-1}·K^{-1})**	**$-\Delta G°$ (kcal·mol^{-1})**
G/C	1000	87.2	241	12.5
	420	83.9	232	12.1
	220	81.1	226	11.1
	120	80.7	227	10.2
	30	71.5	206	7.71
D/T	1000	73.6	201	11.2
	420	73.5	203	10.6
	120	69.1	195	8.74
	30	62.0	179	6.45
I/C	1000	83.6	233	11.2
	420	81.4	229	10.3
	120	80.1	231	8.38
	30	77.0	227	6.46
A/T	1000	76.5	213	10.4
	420	70.2	194	9.92
	220	66.7	187	8.81
	120	67.2	191	7.88
	30	60.1	174	6.26
G/T	1000	78.2	223	9.15
	420	72.1	205	8.44
	120	66.1	190	7.06
	30	58.7	172	5.26
I/T	1000	69.6	196	8.83
	420	67.8	192	8.12
	120	58.3	167	6.65
	30	55.3	162	5.09

Table S2. Thermodynamic parameters of DNA duplexes in 3 M ethanol solutions of varying Na^+ concentration [a].

X/Y	[Na^+] (mM)	$-\Delta H°$ (kcal mol^{-1})	$-\Delta S°$ (cal·mol^{-1}·K^{-1})	$-\Delta G°$ (kcal·mol^{-1})
G/C	420	82.2	232	10.1
	220	86.1	247	9.67
	120	80.1	230	8.88
	30	79.4	284	6.89
D/T	420	77.2	220	8.92
	220	85.4	248	8.59
	120	82.0	239	7.94
	30	74.8	222	5.96
I/C	420	85.0	246	8.68
	220	86.9	253	8.38
	120	87.3	257	7.56
	30	83.6	251	5.70
A/T	420	74.1	212	8.38
	220	77.2	223	8.12
	120	66.7	191	7.48
	30	72.3	214	5.83
G/T	420	78.9	231	7.36
	220	75.8	222	6.96
	120	80.1	239	6.17
	30	72.2	218	4.72
I/T	420	70.4	204	7.13
	220	78.9	233	6.66
	120	80.8	239	5.88
	30	69.6	210	4.49

Note: [a] Data in 1 M Na^+ were unavailable because these conditions resulted in a non-two-state transition.

Table S3. Thermodynamic parameters of DNA duplexes in solutions of varying Mg^{2+} concentration.

X/Y	$[Mg^{2+}]$ (mM)	$-\Delta H°$ (kcal·mol^{-1})	$-\Delta S°$ (cal·mol^{-1}·K^{-1})	$-\Delta G°$ (kcal·mol^{-1})
G/C	40	96.7	272	12.5
	10	93.5	263	11.9
	4	88.5	250	11.1
	1	87.7	253	9.29
D/T	40	85.8	241	11.2
	10	85.0	241	10.3
	4	71.4	200	9.52
	1	69.5	199	7.93
I/C	40	99.1	283	11.3
	10	84.3	239	10.3
	4	84.1	241	9.48
	1	83.4	244	7.88
A/T	40	89.5	255	10.5
	10	80.5	228	9.75
	4	73.9	210	8.89
	1	67.2	193	7.48
G/T	40	85.6	248	8.80
	10	76.6	220	8.32
	4	73.3	212	7.61
	1	64.2	187	6.27
I/T	40	83.0	241	8.38
	10	75.4	218	7.90
	4	71.6	207	7.31
	1	60.1	175	5.97

Figure S1. (**a**) CD spectra of d(TTTGTATCXCAAT)/d(ATTGYGATACAAA) (X/Y = G/C, I/C, D/T, A/T, G/T, or I/T) at 1 M Na^+ and 5 °C; (**b**) CD spectra of d(TTTGTATCACAAT)/d(ATTGTGATACAAA) at 1 M Na^+ (red), 30 mM Na^+ (black), or 1 M Na^+ in 3 M ethanol solution (blue) at 5 °C or 70 °C.

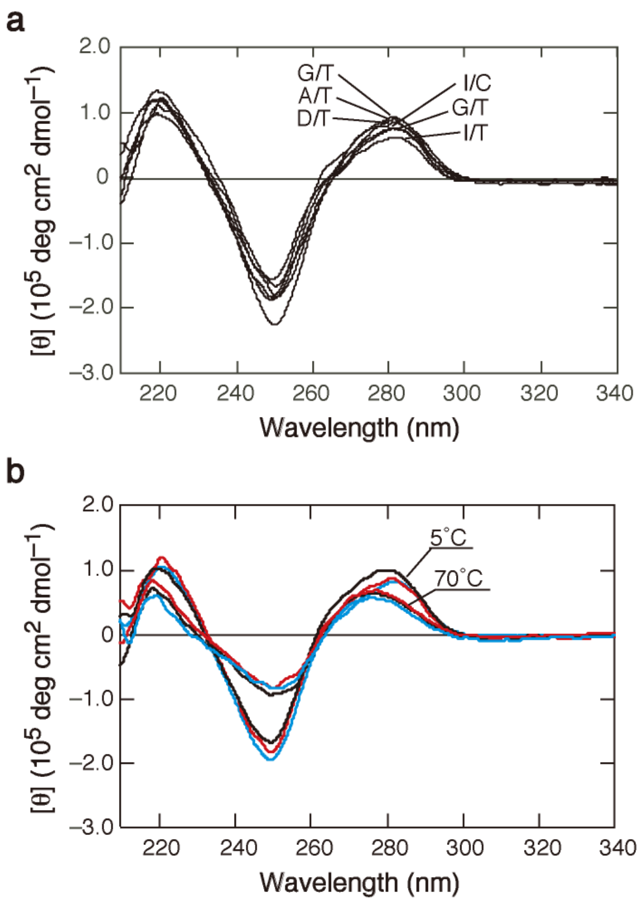

Figure S2. The correlations between values of the slope and intercept of the linear regression of $-\Delta G°$ versus log [M] plots of the 13-mer duplexes in solutions containing Na^+ (black), Na^+ with 3 M ethanol (red), or Mg^{2+} (purple).

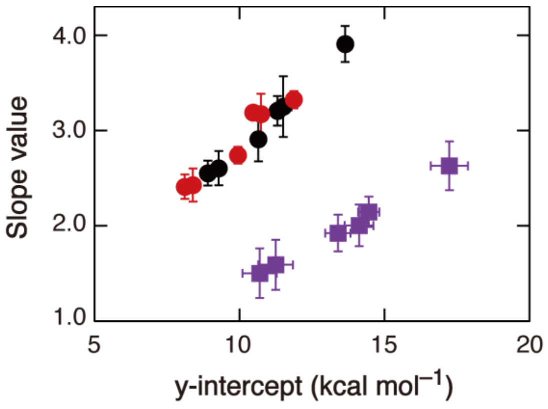

Supplement: Supplementary File 1 [file molecules-19-11613-s001.pdf]
